# Metabolic effects of a mitochondrial‐targeted coenzyme Q analog in high fat fed obese mice

**DOI:** 10.1002/prp2.301

**Published:** 2017-03-10

**Authors:** Brian D. Fink, Deng Fu Guo, Chaitanya A. Kulkarni, Kamal Rahmouni, Robert J. Kerns, William I. Sivitz

**Affiliations:** ^1^ Department of Internal Medicine/EndocrinologyUniversity of Iowa and the Iowa City Veterans Affairs Medical CenterIowa CityIowa52242; ^2^ Department of PharmacologyUniversity of IowaIowa CityIowa52242; ^3^ Department of Pharmaceutical Sciences and Experimental TherapeuticsUniversity of IowaIowa CityIowa52242; ^4^ Departments of Pharmacology and Internal MedicineUniversity of IowaIowa CityIowa52242

**Keywords:** Antioxidants, coenzyme Q, leptin, mitochondria, neuropeptide Y, Obesity

## Abstract

We recently reported that mitoquinone (mitoQ, 500 *μ*mol/L) added to drinking water of C57BL/6J mice attenuated weight gain, decreased food intake, increased hypothalamic orexigenic gene expression, and mitigated oxidative stress when administered from the onset of high‐fat (HF) feeding. Here, we examined the effects of mitoQ on pre‐existing obesity in C57BL/6J mice first made obese by 107 days of HF feeding. In contrast to our preventative study, we found that already obese mice did not tolerate mitoQ at 500 *μ*mol/L. Within 4 days of administration, obese mice markedly decreased food and water intake and lost substantial weight necessitating a dose reduction to 250 *μ*mol/L. Food and water intake then improved. Over the next 4 weeks, body mass of the mitoQ‐treated mice increased faster than vehicle‐treated controls but did not catch up. Over the subsequent 10 weeks, weights of the mitoQ‐treated group remained significantly less than vehicle control, but percent fat and food intake did not differ. Although the mitoQ‐treated groups continued to drink less, there was no difference in percent body fluid and no laboratory evidence of dehydration at study end. At the time of killing, hypothalamic NPY gene expression was reduced in the mitoQ‐treated mice . Liver fat was markedly increased by HF feeding but did not differ between mitoQ and vehicle groups and, in contrast to our previous preventative study, there was no improvement in plasma alanine amino transferase or liver hydroperoxides. In summary, administration of mitoQ to already obese mice attenuated weight gain, but showed limited overall benefit.

**Abbreviations**
AgRP: Agouti‐related peptide
ALT: alanine aminotransferase
CART: cocaine and amphetamine regulated transcript
CNS: central nervous system
CoQ: coenzyme Q
DHPA: 10‐acetyl‐3,7‐dihydroxyphenoxazine
HF: high fat
LepRb: leptin receptor, long form
MitoQ: mitoquinone
NF: normal fat
NMR: nuclear magnetic resonance
NPY: neuropeptide Y
POM: proopiomelanocortin
ROS: reactive oxygen species
TPP^+^: tetraphenylphosphonium
*V*O_2_: oxygen consumption
Δ*Ψ*: mitochondrial inner membrane potential

## Introduction

Obesity is a well‐recognized worldwide epidemic associated with multiple co‐morbidities and commonly resistant to efforts at lifestyle modification (Apovian 2016). Available drug therapy is, at best, only partially effective and associated with side effects; thus, warranting continued efforts to develop new pharmacologic approaches. It has long been known that chemical induction of mitochondria respiratory uncoupling, a means to direct stored energy towards heat production rather than anabolic processes, is an effective means of inducing weight loss (Cutting et al. 1933; Perry et al. 2015). However, the degree of uncoupling can be substantial and human administration has resulted in severe and unacceptable, life‐threatening toxicity (Perry et al. 2015). On the other hand, given that only sight degrees of daily energy dissipation, over long periods of time can lead to significant weight loss, there has been recent interest in developing safe agents that induce only mild respiratory uncoupling (Harper et al. 2001; Geisler 2011; Thrush et al. 2013).

**Figure 1:**
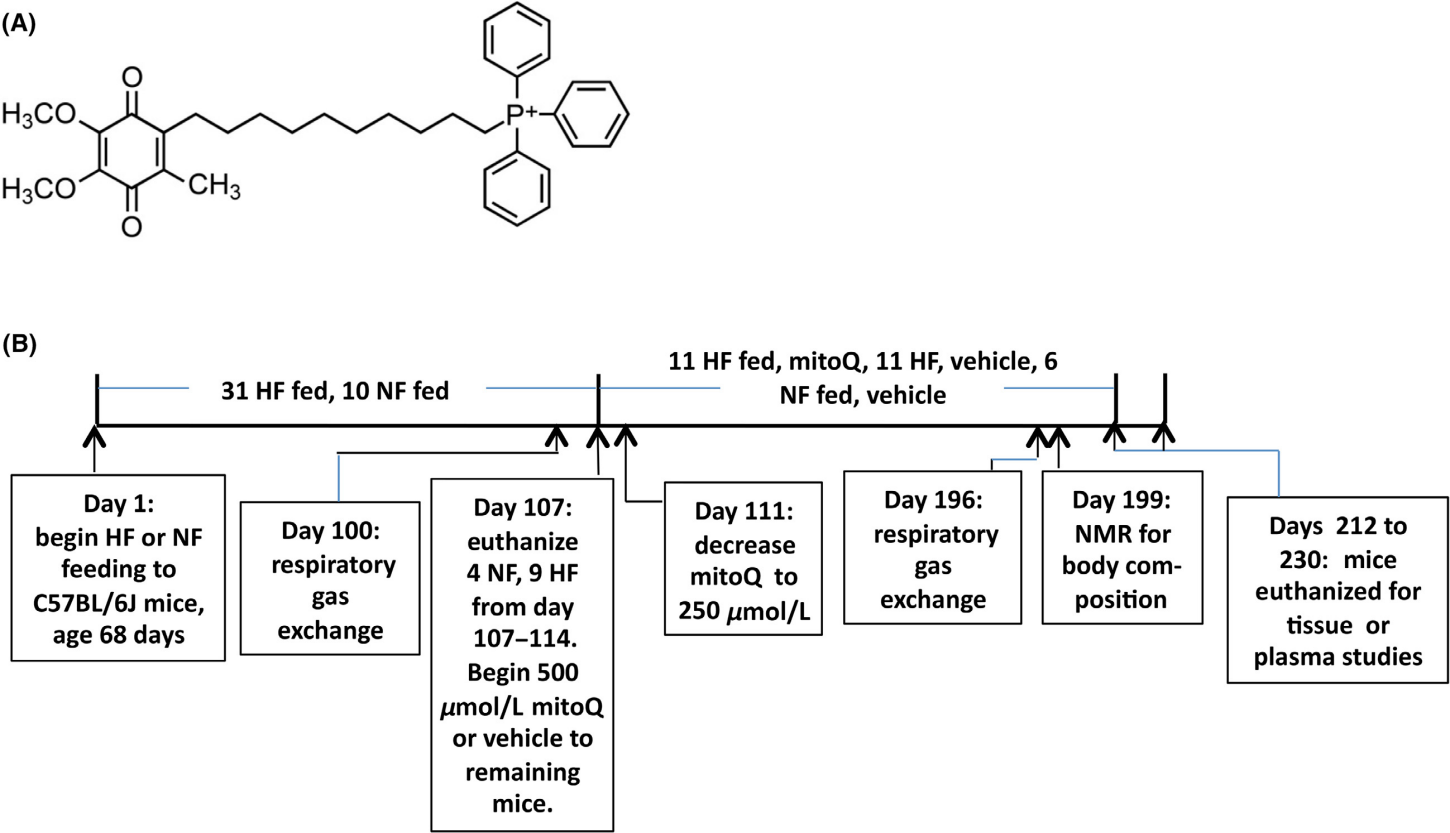
MitoQ structure and study protocol. (A) Structure. (B) Protocol indicating times of treatments and procedures. Mice were fed normal chow (NF) or high fat (HF) from days 1 to 107. After day 107, HF‐fed mice were either killed for baseline study or treated with mitoQ or vehicle and continued on HF until study end. NF‐fed mice were either killed or treated with vehicle and continued on NF. At baseline and study end, mice were killed over the days indicated in staggered fashion so that the average number of days treated did not vary between diet or treatment groups. Data represent mean ± SE. HF, High fat; MitoQ, mitoquinone.

Mitoquinone (mitoQ, Fig. 1A) is a derivative of endogenous coenzyme Q (CoQ) consisting of the quinone moiety and a saturated ten carbon side chain as opposed to the 50 carbon unsaturated side chain of CoQ (James et al. 2005). A cationic moiety, triphenylphosphonium (TPP^+^), is appended to the end of the side chain in order to target mitochondria, given the relative negative charge within the mitochondrial matrix. MitoQ was developed as an antioxidant compound and has been well documented to act in this fashion by virtue of its capacity to block lipid peroxidation (Kelso et al. 2002). However, we recently found that mitoQ, when administered to bovine aortic endothelial cells, had bioenergetic activity as an uncoupler of mitochondrial respiration (Fink et al. 2012). This led us to test the hypothesis that mitoQ might prevent weight gain in mice susceptible to obesity. Indeed, we recently administered mitoQ to obesity prone C57BL/6J mice at the onset of high fat (HF)‐feeding and reported that the drug decreased weight gain and fat mass and reduced hepatic oxidative damage due to steatosis (Fink et al. 2014).

In this study, we administered mitoQ to C57BL/6J mice already obese by antecedent HF feeding in an attempt to ameliorate or reverse pre‐existing obesity.

## Materials and Methods

### Reagents and supplies

Synthesis of MitoQ mesylate and preparation of the cyclodextrin complex of MitoQ mesylate are described in detail in supporting information. All other reagents, kits, and supplies were as specified or purchased from standard sources.

### Animal procedures

Male C57BL/6J mice were obtained from Jackson Laboratories (Bar Harbor, ME). Mice were fed a normal rodent diet (13% kcal fat, diet 7001, Teklad, Harlan Labs, Madison, WI) until initiation of the dietary protocol at age 68 days and maintained according to National Institute of Health guidelines. The protocol was approved by our institutional Animal Care and Use Committee.

The protocol and procedures are depicted in Figure 1B. At 68 days of age, mice were either continued on the normal fat (NF) diet or begun on a HF diet (lard, 60% kcal fat, D12492, Research Diets, New Brunswick, NJ). After 107 days on these dietary regimens, some of the NF and HF mice were killed for baseline data (Fig. 1B). The remaining HF‐fed mice were treated either with mitoQ (500 *μ*mol/L) as the mesylate salt complexed with cyclodextrin or vehicle (cyclodextrin alone) added to the drinking water. The remaining NF‐fed mice were treated with vehicle added to the drinking water. Mice were randomly assigned to these treatment groups after stratification for body mass. After 4 days, the mitoQ concentration in the drinking water was reduced to 250 *μ*mol/L (with an equivalent decrease for vehicle groups) because of a rapid drop in water and food intake (see results) concerning for animal welfare. Within each treatment group (Fig. 1B), two mice were housed per cage except for a single mouse in one cage for each group with an odd number of mice. Mice were weighed every 6‐7 days (except at 4 days after initiation of mitoQ) and food and water intake determined by the difference between the added and remaining supply. Mice were killed between days 214 and 230 with isoflurane for collection of blood by cardiac puncture and isolation of liver and hypothalamic tissue.

### Hypothalamic gene expression

Whole hypothalami were dissected free and immersed in liquid nitrogen for 5 min prior to storage at −80°C. Total RNA was isolated using RNeasy Plus Mini Kit from Qiagen. Five μg of total RNA in final volume of 100 *μ*L were used to synthesize first‐strand cDNAs with the Super‐Script pre‐amplification system. Then, 10 *μ*L of cDNA and 0.4 mmol/L of primers were added in a final volume of 25 *μ*L PCR mixture (iQ SYBR Green supermix, Bio‐Rad, Hercules, California USA), and amplified in an iQ5 Multicolor Real Time PCR Detection System (Bio‐Rad). The PCR conditions for all genes were as follow: denaturation for 5 min at 95°C, then 40 cycles for 30 sec at 95°C and 30 sec at 60°C. *β*‐actin RNA expression was used as internal control to normalize mRNA expression of these genes. The primer set for each gene are: Agouti‐related peptide (AgRP), CAGAAGCTTTGGCGGAGGT (Sense), AGGACTCGTGCAGCCTTACAC (Anti‐sense); Cocaine‐ and amphetamine‐regulated transcript (CART), ATGGAGAGCTCCCGCCTG (Sense), CAGCTCCTTCTCGTGGGAC (Anti‐sense); Neuropeptide Y (NPY), TCAGACCTCTTAATGAAGGAAAGCA (Sense), GAGAACAAGTTTCATTTCCCATCA (Anti‐sense); proopiomelanocortin (POMC), CTGCTTCAGACCTCCATAGATGTG (Sense), CAGCGAGAGGTCGAGTTTGC (Anti‐sense); the long form of the leptin receptor (LepRb), TGTTTTGGGACGATGTTCCA (Sense), GCTTGGTAAAAAGATGCTCAAATG (Anti‐sense); and *β*‐actin (CATCCTCTTCCTCCCTGGAGA (sense), TTCCATACCCAAGAAGGAAGG (anti‐sense).

### Leptin concentrations

Plasma leptin was determined using a mouse leptin ELISA Kit (Sigma Aldrich). Plasma from the NF groups required no dilution. Plasma samples from the HF groups were diluted 1:8 to avoid exceeding the standard curve.

### Body composition

Total body, fat, lean, and fluid mass were determined by nuclear magnetic resonance (NMR) spectroscopy using a Bruker mini spec LF 90II instrument. To analyze body composition, mice were placed into a restraint tube and inserted into the rodent‐sized NMR apparatus adjusting the volume of the chamber based on the size of the animal.

### Isolation of mitochondria

Liver mitochondria were prepared by differential centrifugation as we described previously (O'Malley et al. 2006; Fink et al. 2009). In addition, mitochondria were purified on a self‐generating Percoll^®^ (Sigma) gradient using a Beckman XL‐80 ultracentrifuge and SW60 swinging bucket rotor. Mitochondria were suspended in isolation medium (0.25 mol/L sucrose, 5 mmol/L HEPES pH 7.2, 0.1 mmol/L EDTA, 0.1% defatted bovine serum albumin), layered over a solution of 3 parts Percoll/7 parts isolation medium, and centrifuged at 4°C for 30 min at 90,000*g*. The purified mitochondrial band near the bottom of the tube was transferred to 1.5 mL centrifuge tubes containing isolation medium lacking BSA, spun at 4°C in a microfuge at 8500*g* for 5 min, and the pellet washed a second time. The protein content of the final suspension was determined using the method of Bradford.

### Generation of the 2‐deoxyglucose ATP energy clamp

We used a novel method that we recently described (Yu et al. 2013) to carry out bioenergetic studies of isolated mitochondria under conditions of clamped ADP and membrane potential (ΔΨ). Studies were carried out in the presence of excess 2‐deoxyglucose (2DOG) and hexokinase (HK) and varying amounts of added ADP or ATP. ATP generated from ADP under these conditions drives the conversion of 2DOG to 2DOG phosphate (2DOGP) while regenerating ADP. The reaction occurs rapidly and irreversibly, thereby effectively clamping ADP concentrations and, consequently, also clamping Δ*Ψ* dependent on the amount of exogenous ADP or ATP added. This technique enabled bioenergetic studies to be carried out over respiratory states ranging from state 4 (no added ADP) to state 3 (ADP added in different amounts). For this method to be effective, membrane potential should decrease in stepwise fashion to plateau levels with each incremental addition of ADP (or ATP), which was the case, as we have shown in the past for muscle (Yu et al. 2013), liver (Yu et al. 2014), and heart (Yu et al. 2014) mitochondria.

### Respiration and membrane potential

Respiration and ΔΨ were determined using an Oxygraph‐2k high resolution respirometer (Oroboros Instruments, Innsbruck, Austria) fitted with a potential‐sensitive TPP^+^ electrode. Mitochondria (0.1 mg/mL) were fueled by combined substrates consisting of 5 mmol/L succinate + 5 mmol/L glutamate + 1 mmol/L malate and incubated at 37°C in 2 mL of ionic respiratory buffer (105 mmol/L KCl, 10 mmol/L NaCl, 5 mmol/L Na_2_HPO_4_, 2 mmol/L MgCl_2_, 10 mmol/L HEPES pH 7.2, 1 mmol/L EGTA, 0.2% defatted BSA) with 5 U/mL hexokinase (Worthington Biochemical), and 5 mmol/L 2‐deoxyglucose. ADP was added sequentially to achieve the desired final concentrations with plateaus in respiration and potential achieved after each addition. A TPP^+^ standard curve was performed in each run by adding tetraphenylphosphonium chloride at concentrations of 0.5, 1.0, 1.5, and 2.0 *μ*mol/L, prior to the addition of mitochondria to the chamber.

### Hepatic lipid extraction and liver hydroperoxide determination

Lipid hydroperoxides contained in total hepatic extracts from whole liver tissue were quantified using a commercially available lipid hydroperoxide assay kit (Cayman Chemical) according to the manufacturer's instructions. To extract lipids, portions of liver tissue were removed immediately after killing, weighed (average weight, 0.16 g) and immersed in liquid nitrogen for 5 min prior to storage at −80°C. Frozen tissue samples were then placed in glass tubes containing 0.25 mL of water, homogenized for 30 sec using an Omni TH hand held tissue homogenizer, extracted using chloroform/methanol, and assayed for lipid hydroperoxides.

To determine hepatic total lipid content, 0.5 mL of lipid extracts were placed in microfuge tubes and evaporated overnight for complete solvent removal. Lipid weight was determined as the difference in tube weight before addition of extract and after solvent evaporation.

### Liver Histology

Liver tissue for lipid demonstration was frozen in Optimal Cutting Temperature (OCT) Compound (Sakura Finetek, Torrance, CA), and sectioned with a cryostat at 10 *μ*mol/L thickness. Frozen slides were then fixed in formalin vapors at 50°C for 15 min. After a brief rinse in distilled water to eliminate the OCT compound, the slides s were stained with hematoxylin for 30 sec, rinsed for 5 min in running tap water, and then placed in Oil‐Red‐O solution for 15 min. The sections were quickly rinsed in distilled water and cover slipped in an aqueous mounting media (Faramount, Dako #S3025) and visualized using an Olympus BX‐61 light microscope.

### Indirect calorimetry (whole animal gas exchange)

This was accomplished using a PhysioScan Metabolic System (Omnitech Electronics) to assess gas exchange in small animals. Mice were placed within the chamber for 20 min. Gas exchange was determined over the last 5 min at which time oxygen consumption (*V*O_2_) had reached a steady plateau in the resting animal.

### Serum chemistry

Serum alanine aminotransferase (ALT), sodium, and urea nitrogen were performed by the clinical chemistry laboratory of the Iowa City VA Medical Center by automated methods. ALT was done by coupling the ALT dependent reaction between L‐argine and 2‐oxoglutarate to NADH oxidation by pyruvate and lactate dehydrogenase. Sodium was determined using an ion‐selective electrode. Urea nitrogen was determined by urease catalyzed NH_4_^+^ formation and coupling to NADH reduction by 2‐oxoglutarate and glutamate dehydrogenase.

### Statistics

Data were analyzed by 2‐factor ANOVA (drug x diet), one way ANOVA, or two‐tailed, unpaired *t*‐test as indicated in the figures and legends. Significance required *P* at least <0.05. We performed the same analyses (data not shown) after excluding the mice placed in single cages (one each in the HF‐fed vehicle and HF‐fed mitoQ groups killed at study end and one in the HF group killed at day 107). All data remained significant or nonsignificant as stated for inclusion of all mice.

## Results

### Body weight and composition

**Figure 2:**
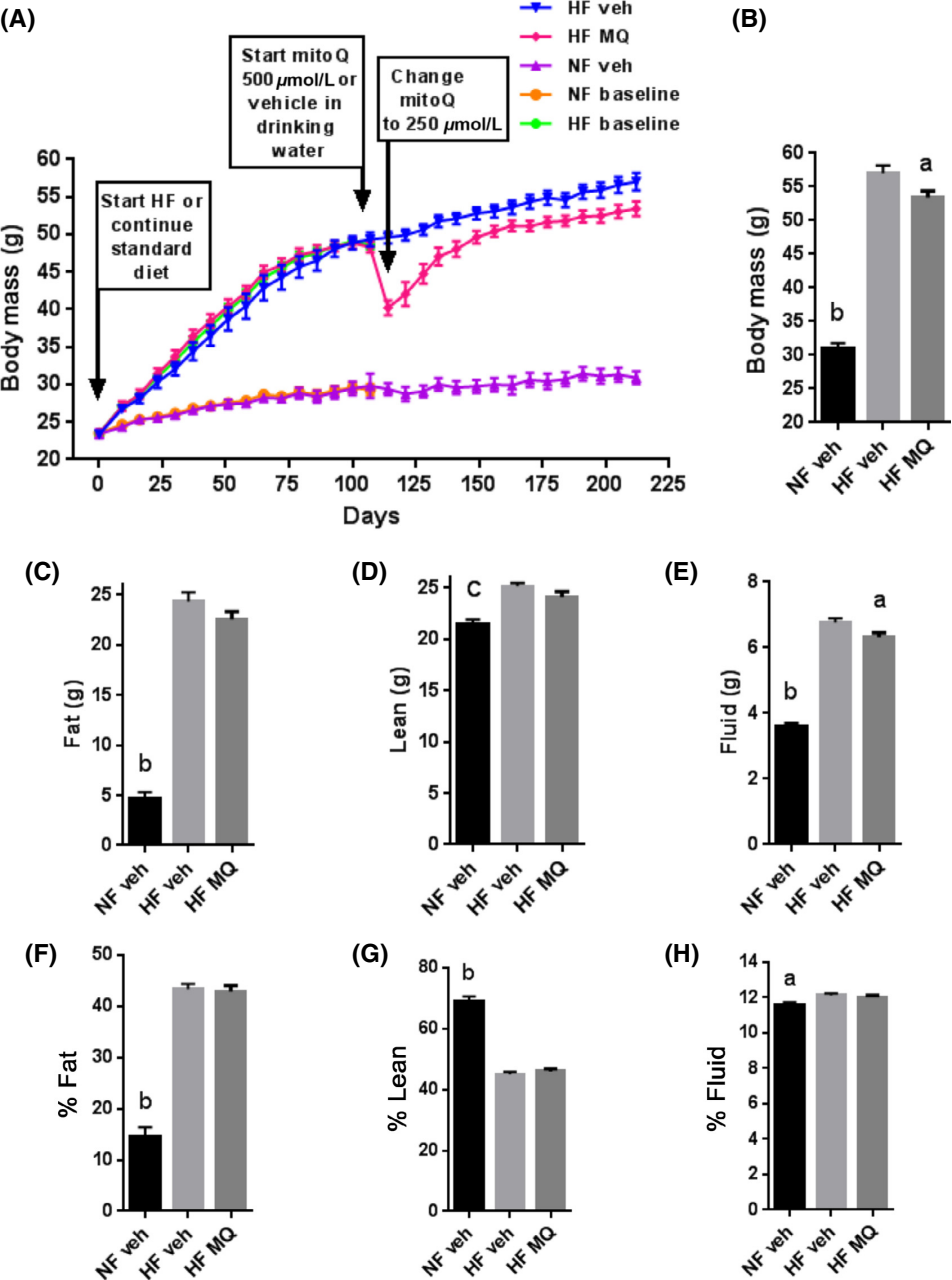
Body mass and composition. (A) Change in body mass over time in high fat vehicle‐treated (HF veh), HF mitoQ‐treated (HF MQ), normal fed vehicle‐treated (NF veh), and in mice killed after 107 days fed normal fat (NF baseline) or high fat (HF baseline) groups. (B) Body weight at study end (day 212) in HF veh, HF MQ, and NF veh groups. (C–E) Body composition expressed in grams of fat, lean, and fluid mass, respectively, determined by NMR at day 199. (F–H) Body composition expressed as % fat, % lean, or % fluid mass determined by NMR. Data represent mean ± SE. *n* = 11 for HF veh and HF MQ mice, *n* = 6 for NF veh mice. ^a^ denotes *P *<* *0.05 compared to HF veh, ^b^denotes *P *<* *0.001 compared to HF veh and to HF MQ mice, ^c^denotes *P *<* *0.01 compared to HF veh and to HF MQ mice by one‐way ANOVA and Tukey's multiple comparison test. HF, High fat; NMR, nuclear magnetic resonance.

As expected, body weight increased markedly compared to controls after 107 days of HF feeding (Fig. 2A). At that point, some NF and HF mice were killed for baseline parameters (Fig. 1B). Other HF mice were assigned to treatment with mitoQ or vehicle administered for the next 107 days (until day 214) while some NF mice were followed on the same normal rodent diet (Fig. 1B). 4 days of mitoQ treatment (500 *μ*mol/L in the drinking water) to HF‐fed mice induced marked weight loss associated with substantially decreased food and water intake, raising animal safety concerns and necessitating a reduction in the concentration of mitoQ to 250 *μ*mol/L. Over the first 21 days following dose reduction, the mitoQ‐treated mice gained at a faster rate than the vehicle‐treated control group. Thereafter, the mitoQ group continued to gradually gain weight but never catching up to the control group and remaining significantly below at study end (Fig. 2B).

Body composition was examined by NMR spectroscopy near study end (Fig. 2C–H). Fat, lean, and fluid mass by NMR of the HF mitoQ and HF vehicle mice were reduced in about the same proportion (Fig. 2C–E). Fluid mass (Fig. 2E) was significantly lower in the HF mitoQ group (6.3%) but actually reduced less than the nonsignificant (due to more variability) decrease in fat mass (7.4%) and to about the same extent as lean mass (6.2%). At study end, there were no differences in body composition measured as % fat, % lean, or % fluid mass between the mitoQ‐treated HF mice and the vehicle‐treated HF‐fed mice (Fig. 2F–H) although fat and lean percent differed greatly compared to NF‐fed mice (Fig. 2F and G).

### Food and water intake

**Figure 3:**
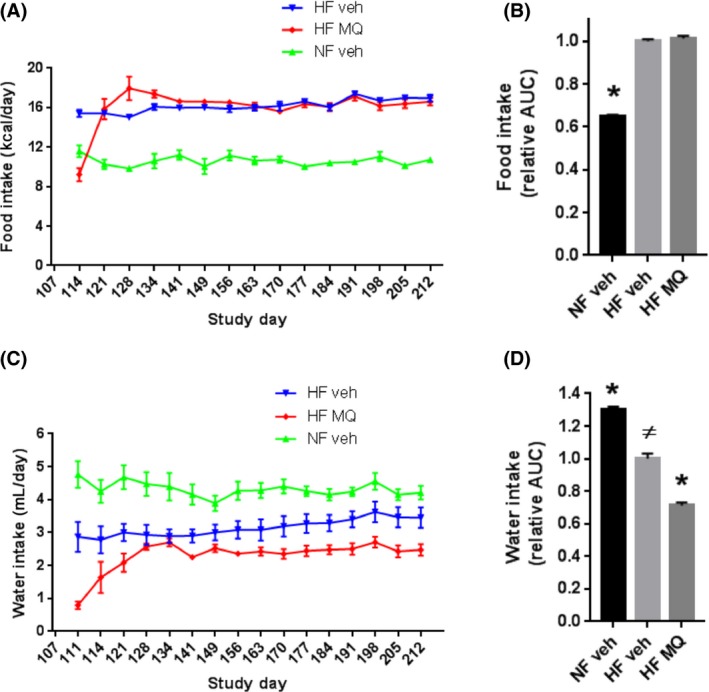
Calorie and water intake indicated by group. (A) Caloric intake (average per cage) measured on study days indicated, beginning on day 114 and determined over intervals since prior *x*‐axis value. (B) Relative area under curve (AUC) (Kcal vs. time) determined from day 128 (stabilization after mitoQ dose reduction) through day 212. (C) Water intake (average per cage) measured on study days indicated beginning on day 111 and determined over intervals since prior *x*‐axis value. (D) Relative area under curve (AUC) (water intake versus time) determined as in panel (C). Data represent mean ± SE. *n* = 6 for HF veh and HF MQ mice, *n* = 3 for NF veh mice. *P <* *0.001 compared to HF veh, ^#^ *P *<* *0.001 compared to NF veh by one‐way ANOVA and Tukey's multiple comparison test. HF, High fat; MitoQ, mitoquinone.

As shown in Figure 3A, food intake (expressed in caloric terms) by HF‐fed mitoQ mice compared to the HF vehicle mice decreased markedly upon starting the higher concentration of mitoQ, but rapidly improved when the mitoQ concentration in the drinking water was reduced, reaching a level transiently higher and then equivalent to the vehicle‐treated, HF‐fed mice. After stabilization, there was no difference in food intake between these groups with both HF groups consuming considerably more calories than the NF mice (Fig. 3B). As shown in Figure 3C, water intake also decreased dramatically upon starting the higher concentration of mitoQ but improved over the next 14–21 days after the mitoQ concentration was reduced. Thereafter, water intake stabilized but did not reach amounts consumed by the vehicle‐treated, HF‐fed mice (Fig. 3D).

### Appetite neuropeptide expression

**Figure 4:**
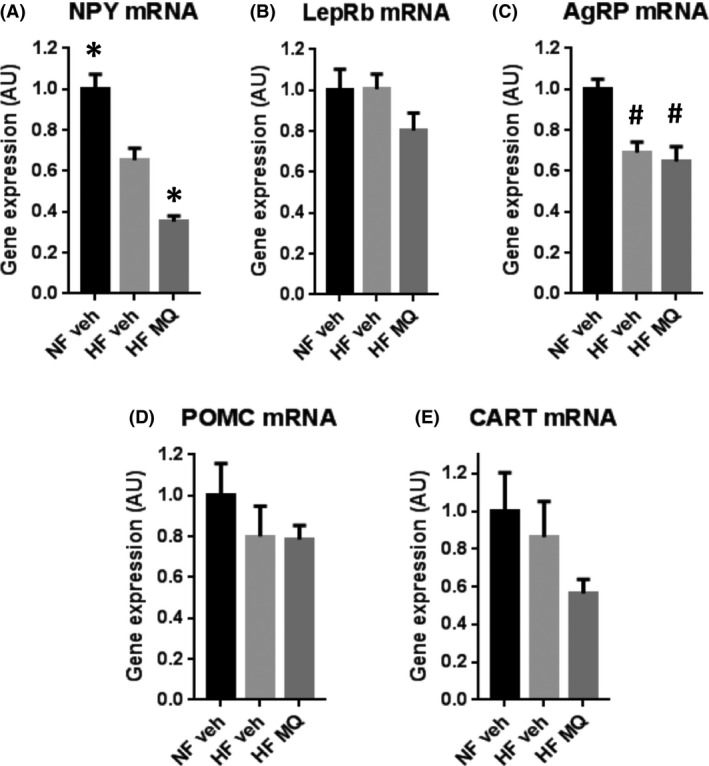
Hypothalamic mRNA expression in NF veh, HF veh, and HF MQ groups. (A) neuropeptide Y; (B) leptin receptor, long form; (C) AgRP; (D) POMC; (E) cocaine and amphetamine regulated transcript. Data represent mean ± SE. *P <* *0.01 compared to HF veh, ^#^ *P *<* *0.01 compared to NF veh by one‐way ANOVA and Tukey's multiple comparison test. *n* = 6 mice per group. HF, High fat.

Hypothalamic mRNA expression of NPY was significantly reduced in the HF mice treated with mitoQ compared to HF‐fed, vehicle‐treated mice (Fig. 4A). No significant changes between these two groups were observed in AgRP, LepRb, CART, or POMC mRNA (Fig 4B–E). Both NPY and AgRP mRNA were lower in both HF groups compared to NF mice (Fig. 4A and C).

### Liver mitochondrial and whole body energetics

**Figure 5:**
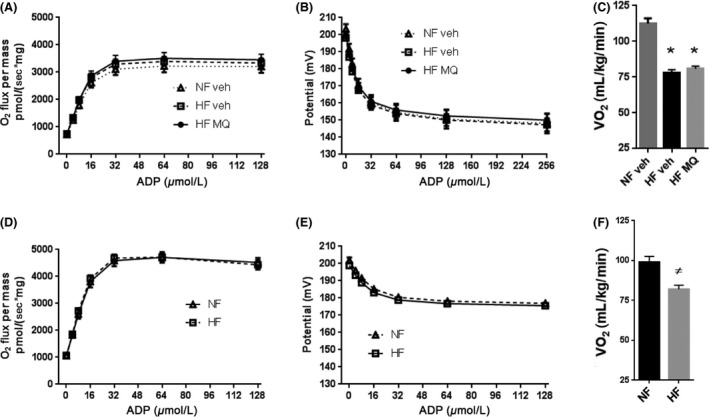
Bioenergetic effects of mitoQ and HF feeding. (A) Respiration at different concentrations of clamped ADP by liver mitochondria isolated at study end from NF veh (*n* = 6), HF veh (*n* = 11), or HF MQ mice (*n* = 11). (B) Inner membrane potential measured simultaneously with respiration in the mitochondria of panel A. (C) Whole body *V*O_2_ measured prior to killing in mice used in panels A and B. (D–E) Corresponding studies of isolated mitochondria from NF (*n* = 4) or HF‐fed mice (*n* = 8) killed after 107 days of diet. (F) Whole body *V*O_2_ measured prior to killing in mice used in panels D and E. Data represent mean ± SE. *P <* *0.001 compared to NF veh by one‐way ANOVA with Tukey's multiple comparison test. #*P *<* *0.01 compared to NF by unpaired, two‐tailed *t*‐test. HF, High fat.

At study end, liver mitochondrial respiration and inner membrane potential were assessed at different levels of clamped ADP availability resulting in respiratory states ranging from states 4 to 3 dependent on the amount of ADP added (Fig. 5). As expected, greater ADP availability resulted in greater respiration and lower membrane potential as charge is utilized by ATP synthase (Fig. 5A and B). However, no differences were observed between HF‐fed mice treated with mitoQ compared to HF‐fed vehicle‐treated mice or to vehicle‐treated mice fed normal rodent chow by two‐way ANOVA (treatment × ADP concentration).

Resting whole body oxygen consumption (*V*O_2_) did not differ between HF‐fed, mitoQ‐treated mice compared to vehicle‐treated, HF‐fed mice (Fig. 5C). As expected, given the known effects of obesity (Sebastian 2013), *V*O_2_ was substantially less in both HF‐fed groups than in NF‐fed mice (Fig. 5C).

We also measured respiration and membrane potential in liver mitochondria isolated from mice killed after 107 days of either NF or HF diets (Fig. 5D–E). HF feeding compared to NF did not affect these parameters by two‐way ANOVA (Fig. 5D and E) but did reduce *V*O_2_ (Fig. 5F). Interestingly, mitochondrial respiration and potential were lower in the mice killed at the study end compared to the mice killed earlier independent of diet (compare Fig. 5A and B to 5D and E), perhaps an effect of aging.

### Hepatic fat content, peroxidation, and function

**Figure 6:**
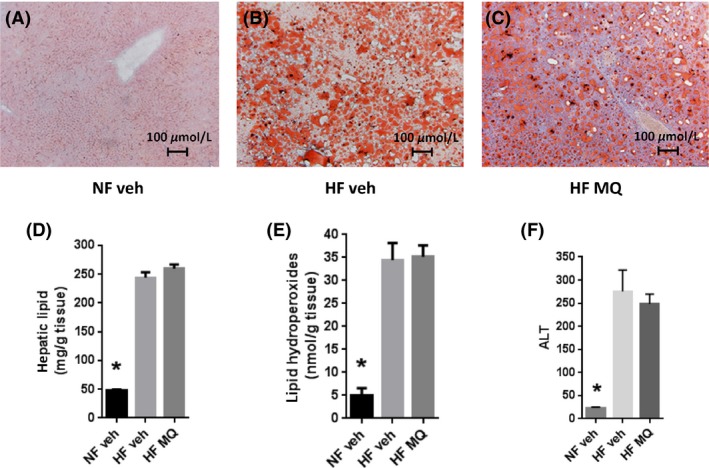
Hepatic lipid, hydroperoxide, and ALT content in mice determined on liver tissue isolated at study end. (A–C) Representative Oil‐Red‐O stained histological images showing liver isolated from NF veh, HF veh, and HF MQ mice, respectively. (D) Hepatic tissue total lipid content in NF veh (*n* = 6), HF veh (*n* = 11) and HF MQ (*n* = 11) mice. (E) Lipid hydroperoxide content in mice of panel D. (F) Plasma ALT in mice of panel D. Data represent mean ± SE. *P <* *0.001 compared to HF veh and HF MQ groups by one‐way ANOVA with Tukey's multiple comparison test. ALT, alanine aminotransferase; HF, High fat.

Although liver fat was markedly increased by HF feeding, mitoQ treatment did not alter fat content or change lipid hydroperoxide content (Fig. 6 A–E). Likewise ALT was markedly increased by HF feeding but not affected by mitoQ (Fig. 6F).

### Insulin, leptin, and insulin sensitivity

**Figure 7:**
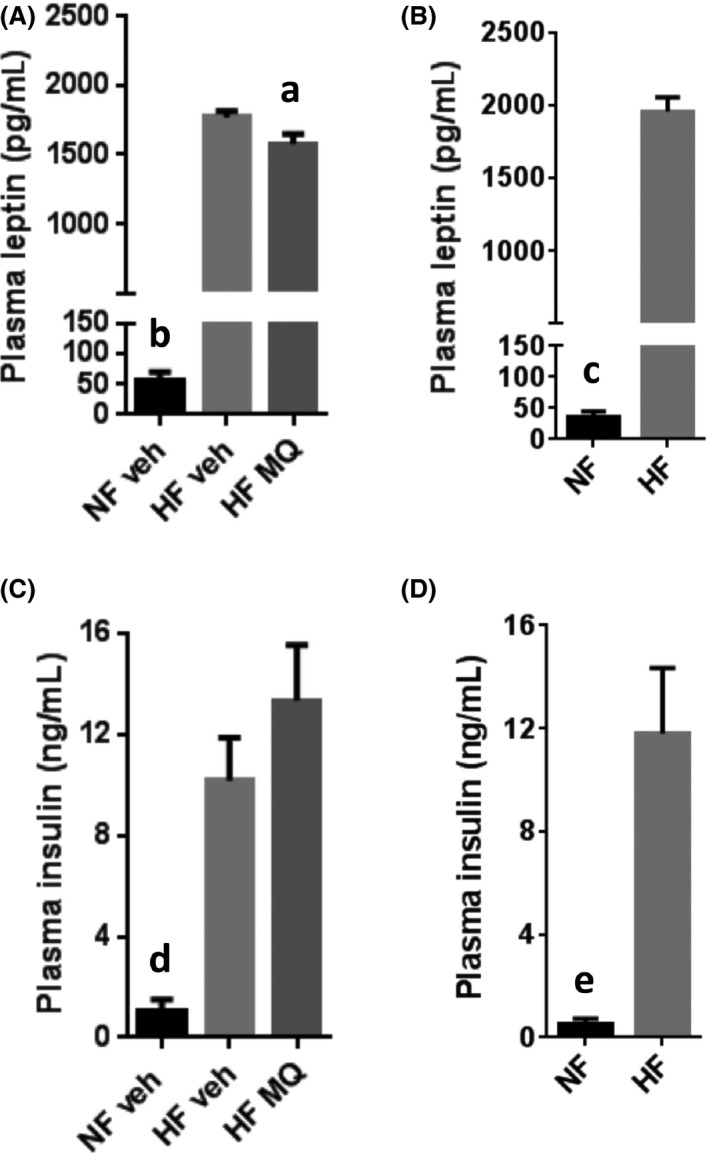
Plasma leptin and insulin concentrations. (A) Leptin in NF veh (*n* = 6), HF veh (*n* = 11), and HF MQ (*n* = 11) mice followed until study end and killed on days 212–230. (B) Leptin in HF and (*n* = 9) NF (*n* = 4) fed mice followed for 107 days and killed on days 107–114. (C and D) Insulin concentrations in mice corresponding to panels A and B. Data represent mean ± SE. ^a^ *P *<* *0.025 compared to HF veh, ^b^ *P *<* *0.001 compared to HF veh and HF MQ mice, ^c^ *P *<* *0.001 compared to HF mice, ^d^ *P *<* *0.025 compared to HF veh and <0.01 compared to HF MQ mice, ^e^ *P *<* *0.025 compared to HF mice. Data in panels A and C were analyzed by one‐way ANOVA with Tukey's multiple comparison test. Data in panels B and D were analyzed by unpaired, two‐tailed *t*‐test. HF, High fat.

Plasma leptin concentrations at study end were significantly lower in the mitoQ‐treated, HF‐fed mice compared to vehicle‐treated, HF‐fed mice (Fig. 7A), while all HF groups had much higher leptin concentrations than NF‐fed mice (Fig. 7A and B). Insulin concentrations did not differ between the mitoQ‐treated HF mice and vehicle‐treated HF mice at study end (Fig. 7C). As expected, HF‐treated groups had much higher insulin concentrations compared to NF (Fig. 7C and D).

### Sodium, potassium, and renal function

**Figure 8:**
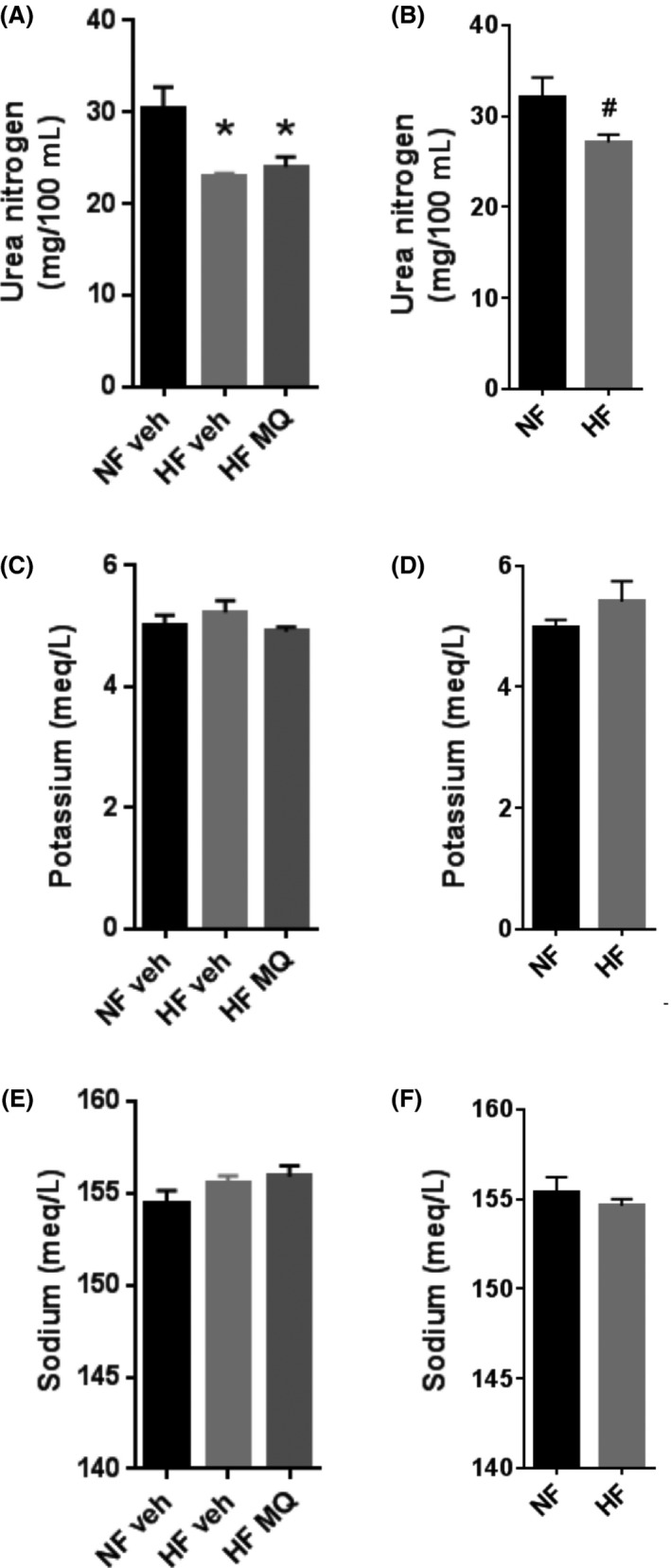
Plasma urea nitrogen, potassium, and sodium concentrations as markers of renal function and hydration. (A) Urea nitrogen in NF veh (*n* = 6), HF veh (*n* = 11), and HF MQ (*n* = 11) mice followed until study end and killed on days 212–230. (B) Urea nitrogen in HF (*n* = 9) and NF (*n* = 4) fed mice followed for 107 days and killed on days 107–114. (C and D) Potassium concentrations in mice corresponding to panels A and B. (E and F) Sodium concentrations in mice corresponding to panels A and B. Data represent mean ± SE. *P <* *0.01 compared to NF veh, ^#^ *P *<* *0.05 compared to NF mice. Data in panels A, C, and E were analyzed by one‐way ANOVA with Tukey's multiple comparison test. Data in panels B, D and F were analyzed by unpaired, two‐tailed *t*‐test. HF, High fat.

Plasma urea nitrogen, potassium, and sodium measured at killing did not differ between mitoQ‐treated and vehicle‐treated, HF‐fed mice measured at study end (Fig. 8). Urea nitrogen was lower in both the mitoQ and vehicle‐treated HF mice compared to NF vehicle‐treated mice (Fig. 8A) and lower in the HF compared to NF mice killed prior to the treatment assignment (Fig. 8B).

## Discussion

In comparison to our prior study (Fink et al. 2014) wherein mitoQ was begun prior to HF feeding, mitoQ was not as well tolerated in mice that were already obese. At a dose of 500 *μ*mol/L in the drinking water, already obese mice manifest a substantial and concerning decrease in food and water intake (Fig. 3A and C). This necessitated a reduction in the concentration to 250 *μ*mol/L after which food and water intake improved. When calculated per amount of water actually consumed after stabilization of water intake (day 128 to study end), the mitoQ dose was 0.62 *μ*mol/L ole per mouse per day. In our prior study at 500 *μ*mol/L in drinking water, the corresponding daily dose was 1.04 *μ*mol/L ole per mouse per day.

We cannot be sure of why mice did not tolerate the higher dose of mitoQ. Taste aversion or general malaise may have been responsible, but we know of no data supporting these possibilities. Other investigators reported transient decreased water intake in normal, non‐obese mice fed mitoQ 500 *μ*mol/L in drinking water (Rodriguez‐Cuenca et al. 2010). This was not severe enough to mandate a change in dose and is consistent with our prior preventative study (Fink et al. 2014) in which we were able to chronically administer this dose. However, in this study of already obese mice, the decrease in food and water intake was enough to raise issues of animal welfare, requiring a change in dosing.

In the first several days after dose reduction, the mitoQ‐treated mice gained weight faster than vehicle controls (Fig. 2A). Thereafter, the rate of gain stabilized, but body mass never caught up to the control mice. Although the mean body mass of the mitoQ group was significantly less by 6.3% than the vehicle‐treated group at study end (Fig. 2B), there was no difference in percent fat, percent lean or percent fluid mass (Fig. 2F, G and H). Although total body water was significantly reduced in the mitoQ‐treated mice compared to vehicle (Fig. 2E), the actual reduction (6.7%) in body water (Fig. 2E) was less than the reduction (7.4%) in fat mass (Fig. 2C) (not significant due to more variability) and quite comparable to the reduction (6.3%) in total body mass (Fig. 2B).

It could be argued that we may have simply observed drug toxicity, even on the lower mitoQ dose. However, there is reason to argue otherwise. After dose reduction mitoQ‐treated, HF mice rapidly gained weight and ate more food (Figs. 2A and 3A). Moreover, the rates of change in these parameters, as well as in water intake, transiently exceeded that for vehicle controls (Figs. 2A, 3A, and C) suggesting recovery from toxicity. Also, any potential toxicity of the lower dose of mitoQ was not enough to ultimately limit food intake since, after initial stabilization, food intake did not differ between the HF mitoQ‐ and HF vehicle‐treated mice. It might be that mitoQ actually did not reduce body mass so much as delay weight gain on HF feeding. However, after stabilization, there was no tendency toward catch up by the mitoQ‐treated mice, as would be expected based on body set point dynamics (Leibel et al. 1995). Decreased water intake is also an unlikely explanation for the study end mitigation of body mass, since there was no difference in percent body fluid at killing and no evidence of dehydration as assessed by plasma sodium and urea nitrogen.

Creatinine concentrations at study end (not shown) were all lower than detectable by our clinical laboratory which is as expected for mice, wherein levels are low compared to humans (Wirth‐Dzieciolowska et al. 2009). However, the data do show that mice did not develop renal insufficiency, as that should produce a detectable rise in creatinine (Wirth‐Dzieciolowska et al. 2009). Note also that urea nitrogen is a better marker for hydration than creatinine and is elevated more than creatinine in dehydration (Armstrong et al. 2016). Moreover, although mitoQ compared to vehicle did not alter urea nitrogen, the analyte was lower in HF groups compared to NF (Fig. 8A and B) vehicle, likely due to steatosis with liver dysfunction and less production of urea.

Although we did not see a significant change in body fat or percent fat in this study, we did observe a significant reduction in plasma leptin, a strong marker of fat mass (Considine et al. 1996). We cannot explain the discrepancy except that it could be due to variability in the measurement of either parameter.

We also observed that mitoQ‐treatment of HF‐fed mice compared to vehicle reduced hypothalamic NPY, an orexigenic peptide (Fig. 4). This was likely a primary effect of mitoQ rather than secondary to changes in leptin or insulin. Increased levels of these hormones would reduce NPY. But, as above, leptin was decreased and insulin concentrations did not differ significantly (Fig. 7A and C). In spite of the decrease in NPY, we saw we saw no change in food intake or in the orexigenic peptide, AgRP. So, the significance of the decrease in NPY is uncertain. Aside from increasing appetite, NPY may impair energy utilization by blocking sympathetic nerve activity (Cassaglia et al. 2014), so the reduction in NPY may have favored bioenergetic action and less weight gain. This is consistent with our finding of less weight gain on mitoQ treatment in spite of no difference in food intake. However, we did not detect bioenergetic effects as might manifest in resting VO_2_ or in altered energetics of isolated liver mitochondria. Unfortunately, for practical workflow reasons, we were not able to assess mitochondrial function beyond liver tissue.

In several ways, our current findings contrast with our past observations wherein we administered mitoQ in preventative fashion (Considine et al. 1996), that is, at the onset of HF feeding. In both studies, the mitoQ‐treated HF mice weighed significantly less at study end than the vehicle‐treated, HF‐fed mice (6.3% in this study, 9.4% in the past study). Study duration was 212 days in this study (107 days of HF feeding before 105 days of HF with mitoQ or vehicle) and 167 days in our past study (HF with mitoQ or vehicle throughout). Mice started at about the same age in both studies (68 days in our present and 72 days in our past study). However, in our preventative study, mitoQ significantly reduced fat mass and food intake. MitoQ also significantly reduced hypothalamic AgRP, NPY, and LepRb mRNAs in our preventative study, whereas mitoQ only reduced NPY mRNA in this study. In both studies bioenergetics, as far as could be detected by resting VO_2_ and liver mitochondrial function, were not altered. In this study, we observed no effect of mitoQ on liver fat, liver hydroperoxides, or liver function measured by ALT. In our preventative study, mitoQ also did not change liver fat content, but significantly and substantially reduced liver hydroperoxides (42%) and ALT (54%).

We must acknowledge another difference between our present and past preventative study (Fink et al. 2014). Here we administered mitoQ as the mesylate salt complexed to cyclodextrin added to the drinking water, whereas in the prior study we added mitoQ bromide. However, it is doubtful that the decrease in tolerability in our current is due to this, since the mesylate salt with cyclodextrin has emerged as the apparent preferred method (Rodriguez‐Cuenca et al. 2010; McManus et al. 2011; Mercer et al. 2012; Feillet‐Coudray et al. 2014; Sakellariou et al. 2016) and cyclodextrin is widely used in pharmacologic studies and considered to be well tolerated (McManus et al. 2011).

MitoQ added to drinking water has been used in other mouse studies in varying doses above and below that which we used herein. Using as little as 100 *μ*mol/L, mitoQ mesylate adsorbed to cyclodextrin administered for 6 months reduced cognitive decline and mitigated oxidative stress in a mouse model of Alzheimer's disease (McManus et al. 2011). However, mitoQ mesylate in cyclodextrin added to drinking water at the same dose, 100 *μ*mol/L, for 15 weeks did not attenuate age‐related oxidative damage in muscle of C57BL/6 mice or alter mitochondrial function in permeabilized myofibers (Sakellariou et al. 2016). Rodriguez‐Cuenca et al. (2010) administered MitoQ, 500 *μ*mol/L, in drinking water, as a *β*‐cyclodextrin complex of the methane sulfonate salt to normally fed wild‐type mice for 20–28 weeks. These authors noted no significant effects on body weight, lean or fat mass, or in markers of oxidative damage. Obese or HF‐fed mice were not studied. The authors concluded that mitoQ was safe in normal wild‐type mice. In that study, water intake was reduced in the first week followed by recovery in to levels that did not differ from controls (6.4 mL/day for mitoQ compared to 6.6 mL/day). Mercer et al. (2012) administered mitoQ mesylate as a cyclodextrin complex in water to mice genetically predisposed to atherosclerosis (ATM^+/−^/ApoE^−/−^ mice) and reported that the mitoQ‐treated animals gained less weight, had less hypercholesterolemia, and hypertriglyceridemia, and showed improvements in hyperglycemia and hepatic steatosis. In that study, mitoQ was initiated at 100 *μ*mol/L but gradually increased to 500 *μ*mol/L. Water intake by the mitoQ‐treated mice was nonsignificantly different from controls but appeared to be about 20% less.

In summary, we show that favorable anti‐obesity effects of mitoQ administered to already obese HF‐fed mice were modest at most. Moreover, mitoQ at the dose administered did not protect against liver fat, liver hydroperoxides, or liver dysfunction. Our results have clear implications for future studies of mitoQ and/or related mitochondrial targeted agents under development (Skulachev et al. 2009; Severina et al. 2013). Tolerability is an issue and may be improved by administration apart from the drinking water (although adding the drug to food may confound measuring food intake). Different dosing regimens need to be compared and obesity per se may have implications for tolerability. Food and water intake need monitoring and more detailed studies of energetics are needed. Possibly, higher doses than used herein, administered in tolerable fashion, or treatment earlier in the course of worsening obesity may be needed for substantial bioenergetic and antioxidant effects.

## Author Contribution

*Participated in research design:* Fink, Rahmouni, Kerns, Sivitz. *Conducted experiments:* Fink, Guo, Kulkarni. *Contributed new reagents or analytic tools:* Kulkarni, Kerns. *Performed data analysis:* Fink, Guo, Rahmouni, Sivitz. *Wrote or contributed to the writing of the manuscript:* Fink, Rahmouni, Kerns, Sivitz.

## Disclosure

None declared.

## Supporting information


**Data S1.** Pharmacology, research & perspectives.
