# Supplementary material for: Metabolic effects of a mitochondrial‐targeted coenzyme Q analog in high fat fed obese mice
Source: Pharmacol Res Perspect. 2017 Mar 10;5(2):e00301. doi: 10.1002/prp2.301 (PMC5368965; doi:10.1002/prp2.301)

Supplemental data

Pharmacology, Research & Perspectives

Metabolic Effects of a Mitochondrial-Targeted Coenzyme Q Analog in High Fat Fed Obese Mice

Brian D. Fink, Deng Fu Guo, Chaitanya A. Kulkarni, Kamal Rahmouni, Robert J. Kerns, and William I. Sivitz

SYNTHESIS OF MITOQ MESYLATE AND PREPARTION OF THE β – CYCLODEXTRIN COMPLEX OF MITOQ MESYLATE

General Methods:

All reagents were purchased from commercial sources and used without further purification. Idebenone was from Brookview Scientific, LLC (Carmel, IN). NMR spectra were obtained using a Bruker Ultrashield 300 MHz instrument at ambient temperature. Chemical shifts are reported in parts per million from low to high field and referenced to residual solvent. Standard abbreviations indicating multiplicity are used as follows: m = multiplet, s = singlet. High resolution mass spectrometry (HRMS) was determined using a Waters Q-Tof Premier mass spectrometer with electrospray ionization (ESI). MitoQ mesylate was purified >95%, as determined by analytical high performance liquid chromatography (HPLC). The analytical HPLC analysis was determined using a Shimadzu system equipped with SPDM10A vp photodiode array detector. The system was connected to a Dell Optiplex GX400 PC and controlled by Shimadzu Client/Server Version 7.4 software. A Restek Allure PFP Propyl (150 mm x 4.6 mm) 5 μm column was used as stationary phase, while mobile phase consisted of solvent A (water, 0.1% TFA) and solvent B (acetonitrile, 0.1% TFA). Gradient elution used the following program: from t = 0 min [solvent A (0.95 mL/min), solvent B (0.05 mL/min)] to t = 40 min [solvent A (0.05 mL/min), solvent B (0.95 mL/min)].

Synthesis of MitoQ mesylate:

Methods adapted from previously reported synthesis of MitoQ bromide (S1). Idebenone (**1**) (Scheme S1) (3 g, 8.9 mmol) was dissolved in dichloromethane (50 mL). Triethylamine (2.5 mL, 17.9 mmol) was added to the solution and the resulting mixture stirred in an ice bath for 30 min. Methanesulfonyl chloride (1.4 mL, 18.1 mmol) in dichloromethane (20 mL) was added dropwise and the reaction stirred at room temperature overnight. TLC indicated reaction was complete as observed for disappearance of idebenone. The organic layer was washed with 1 N HCl four times. The organic layer was dried over sodium sulfate and concentrated *in vacuo* to give idebenone mesylate (**2**) in as an orange oil, which was used without further purification. Idebenone mesylate (8.9 mmol, assuming 100 % yield from the previous step) was dissolved in methanol (50 mL) and cooled in an ice-bath under Argon atmosphere. Sodium borohydride (0.84 g, 22.1 mmol) was added to the solution in small portions over 30 min. Effervescence and color change was observed. The reaction was stirred for another 30 min until the solution remains colorless. The reaction was acidified with 1 N HCl and the product extracted with 50 mL chloroform. The organic layer was dried *in vacuo*. The idebenol mesylate (**3**) obtained as a colorless oil was immediately dissolved in dioxane (25 mL). Triphenylphosphine (3.4 g, 13.3 mmol) was added to the solution and the reaction was refluxed under Ar atmosphere for 3 days. The solvent was partially removed under reduced pressure and mitoquinol (**4**) was precipitated by addition of 50 mL diethyl ether. The precipitated product was collected by decantation and further dried under vacuum. The dried precipitate was then dissolved in ethanol and bubbled with air for 48 h to oxidize mitoquinol to mitoquinone. The ethanol was removed *in vacuo* and the product was placed under vacuum overnight to obtain mitoquinone mesylate (**5**) as an orange foam (3.1 g, 51 %). ^{1}H NMR (300 MHz, $CDCl_3$) δ 7.74 (m, 15H), 3.93 (s, 6H), 3.55 (m, 2H), 2.68 (s, 3H), 2.42 (m, 2H), 1.99 (s, 3H), 1.58 (m, 2H), 1.25 (m, 14H). HRMS (ESI) calculated for (M^+) 583.2972, found 583.2991

(S1) 11.Rao, V. A., Klein, S. R., Bonar, S. J., Zielonka, J., Mizuno, N., Dickey, J. S., Keller, P. W., Joseph, J., Kalyanaraman, B., and Shacter, E. (2010) *J Biol Chem* **285**, 34447-34459.

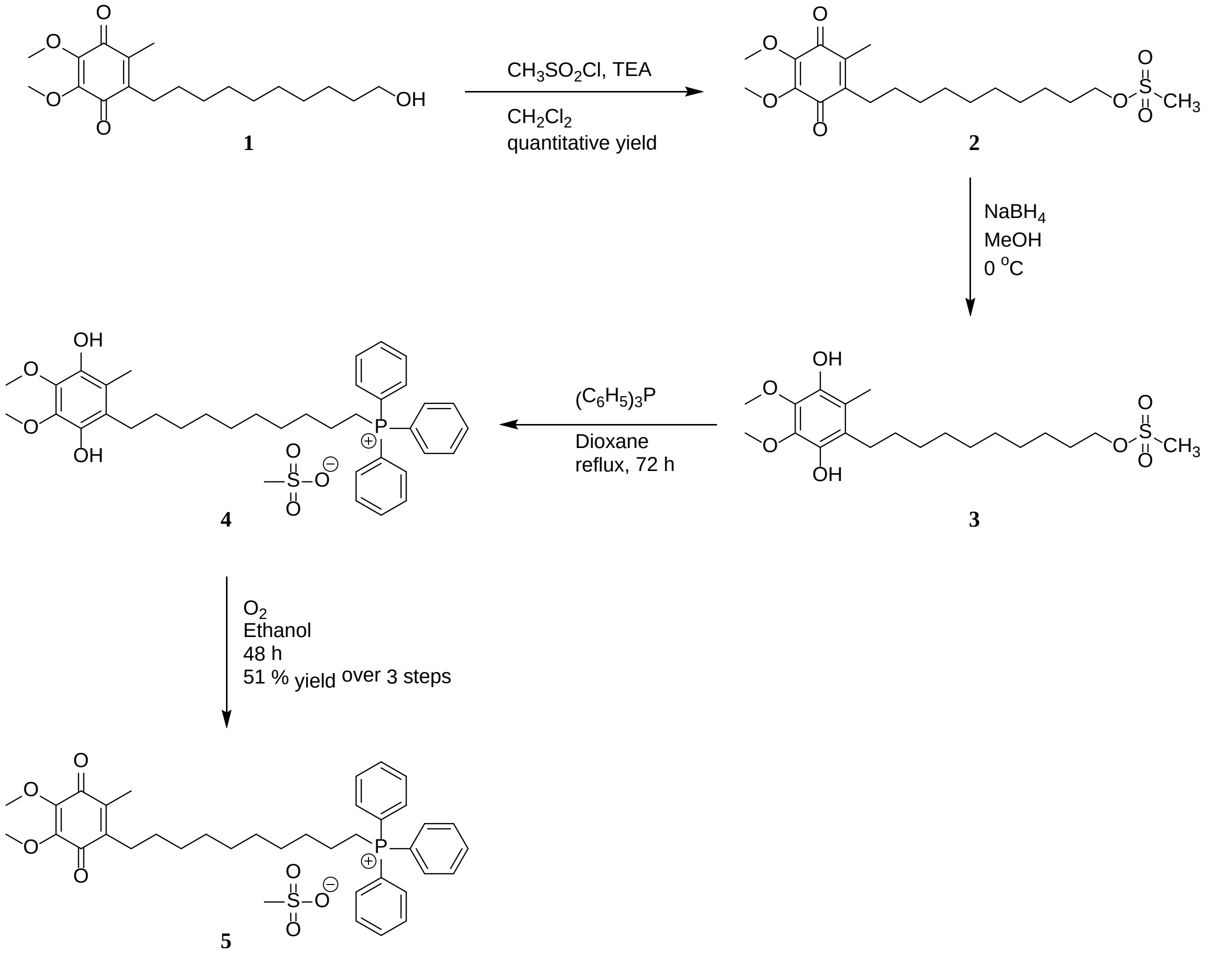

Scheme S1: Synthesis of MitoQ mesylate from commercially available Idebenone.

Preparation of β – cyclodextrin complex of MitoQ mesylate:

Mitoquinone mesylate (1.5 g) was dissolved in nanopure water (25 mL) with warming to 40 ^{o}C to give a clear orange solution. Beta-cyclodextrin (4.5 g) was separately dissolved in nanopure water (75 mL) with heating to 60 ^{o}C to obtain a clear colorless solution. The two solutions were allowed to cool to room temperature, combined and stirred for 1 hour at room temperature. The resulting orange solution was stored in a refrigerator at 5 ^{o}C for 12 hours. The solution was then frozen and lyophilized to a constant weight over 2 days to obtain a yellowish-orange free flowing powder. Based on the ratio of MitoQ mesylate and cyclodextrin used, MitoQ constituted 25 % w/w of the complex (250 mg per gm of the complex). The MitoQ ratio in final powder was further confirmed by establishing a standard curve on analytical HPLC using known concentrations of MitoQ mesylate, then determining the amount of MitoQ mesylate in different samples of the MitoQ mesylate-cyclodextrin complex. The experimentally determined level of MitoQ mesylate in the cyclodextrin complex product was found to be within error of the expected level based on ratio of MitoQ mesylate and β – cyclodextrin used.

^{1}H NMR of MitoQ mesylate (**5**).

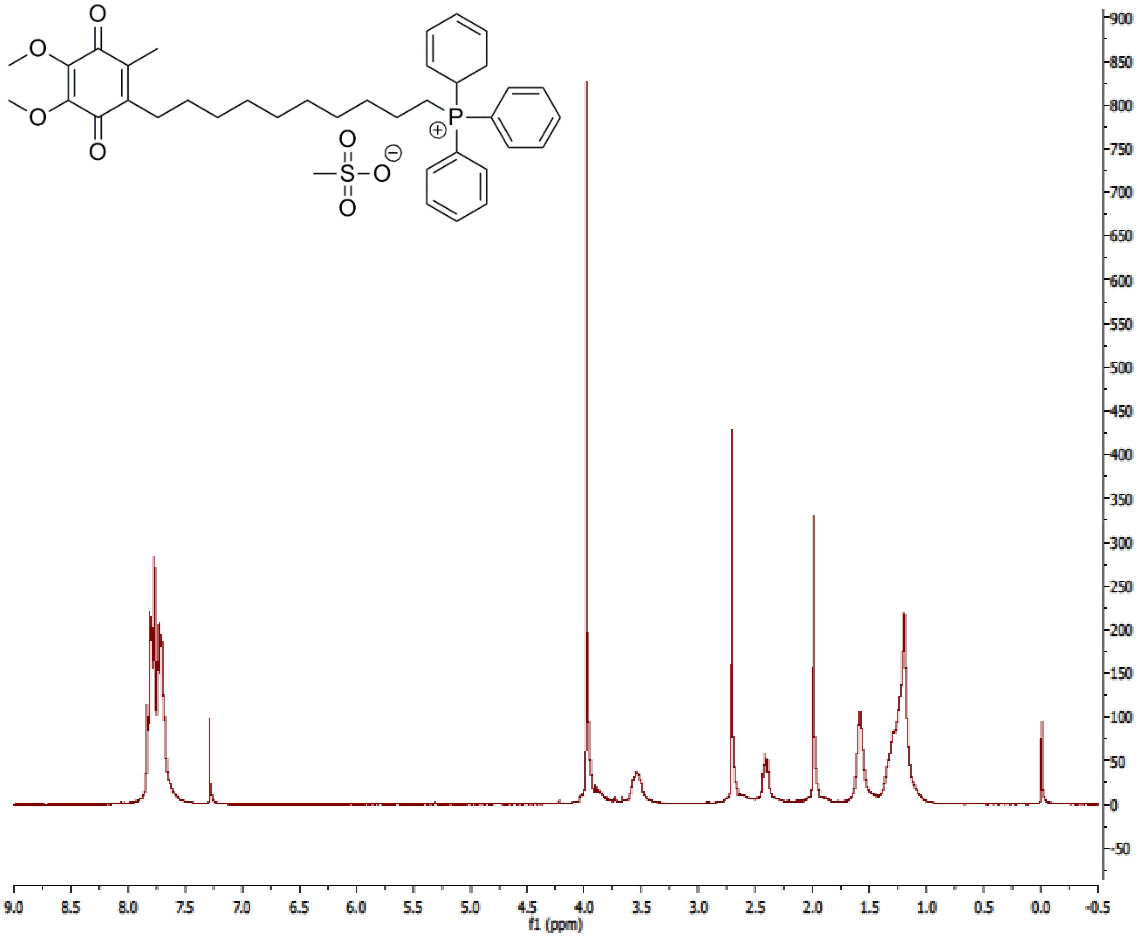

Analytical reversed-phase HPLC of MitoQ mesylate (**5**). Retention time = 32.05 min. Purity >95%. Absorbance wavelength shown = 273 nm.

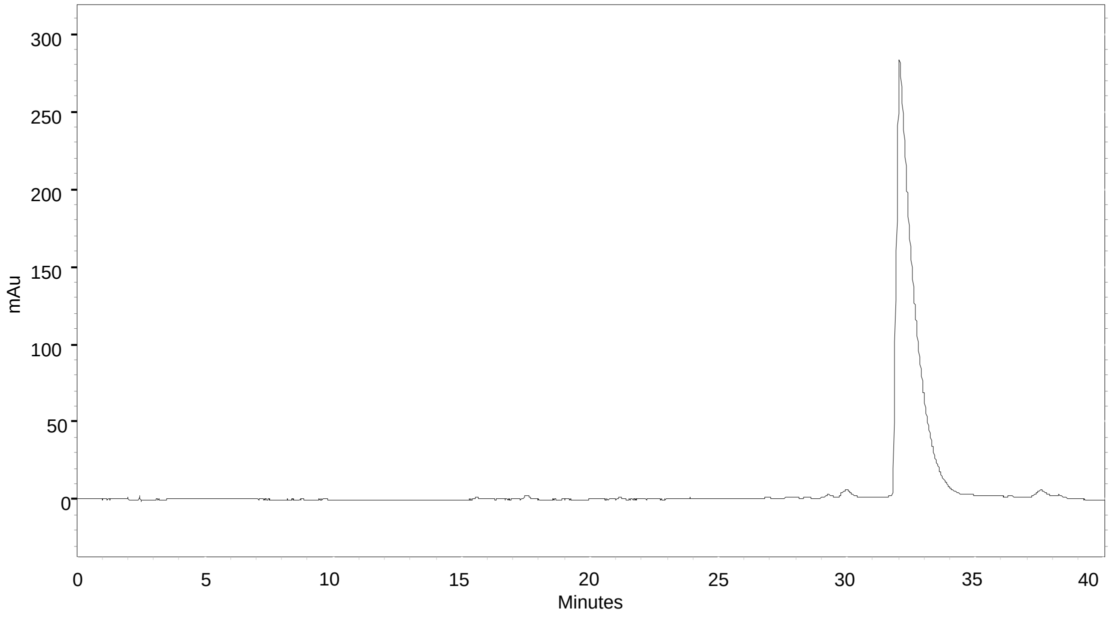

Supplement: Supplementary file 1 — Data S1. Pharmacology, research & perspectives. [file PRP2-5-e00301-s001.pdf]
